# Enhanced heroin analgesic effect in male offspring of sires who self-administered heroin

**DOI:** 10.3389/fphar.2023.1211897

**Published:** 2023-06-14

**Authors:** Wenjing Gao, Tao Pan, Guangyuan Fan, Jian Cui, Tingting Wang, Nan Huang, Changyou Jiang, Lan Ma, Feifei Wang, Xing Liu, Qiumin Le

**Affiliations:** The School of Basic Medical Sciences and Institutes of Brain Science, Fudan University, Shanghai, China

**Keywords:** analgesic activity, heroin self-administration, paternal inheritance, male offspring, cross-sensitization of addictive drugs

## Abstract

**Introduction:** A growing body of evidence suggests that parental substance abuse, even prior to conception, may induce phenotypic changes in offspring. Parental opioid exposure has been shown to affect developmental processes, induce memory deficits, and lead to psycho-emotional disorders in offspring. However, how parental, especially paternal, chronic drug exposure affects offspring remains unexplored.

**Methods:** Adult male rats were subjected to 31 days of heroin self-administration followed by mating with naïve females. Litter size and body weight of F1 offspring were recorded. Object-based attention tests, cocaine self-administration tests, and hot plate tests were used to test for potential effects of chronic paternal heroin seeking on cognition, reward, or analgesic sensitivity in the offspring.

**Results:** Body weight and litter size of the heroin F1 generation were not altered compared to the saline F1 generation. Furthermore, paternal chronic heroin self-administration experience had no significant effect on object-based attention tests or cocaine self-administration behavior in either sex. However, in the hot plate test, although no difference in basal latency was found between the two groups in either sex, a significant increase in the analgesic effect of heroin was observed in the male heroin F1 generation.

**Conclusions:** Taken together, these data provide evidence that paternal chronic heroin self-administration experience could sex-dimorphically increase the analgesic effect of heroin in male offspring, but had no significant effect on response to cocaine reinforcement or attentional behavior.

## Introduction

Accumulating evidence indicates that ancestral environmental insults could influence the developmental process and lead to behavioral changes in the offspring, to adapt to the ever-changing and challenging surrounding environment. Various studies have implicated emotional stimuli, like early life trauma (Gapp et al., 2014; Gapp et al., 2020), stress (Rodgers et al., 2015), depression (Wang et al., 2021), and fear memory (Dias and Ressler, 2014), are a potential trigger for phenotypic variations in future generations. In addition, changes in parental nutritional status, such as high fat diet (Chen et al., 2016), overnutrition or malnutrition (Guillaumin and Peleg-Raibstein, 2023), and high fructose diet (Zou et al., 2023), could induce obesity, metabolic disorders (Zhang et al., 2018), and memory disruptions (Smaga et al., 2023) in the offspring. Parental exposure to toxins, like lipopolysaccharide (Zhang et al., 2021; Carbone et al., 2023) and chemicals (Nilsson et al., 2020; Thorson et al., 2021), could elicit behavioral alterations and deleterious outcomes in the offspring.

In the case of opioid abuse, opioid drugs such as morphine and heroin induce euphoric feelings or alleviate pain at the onset by stimulating opioid receptors (Contet et al., 2004; Wee and Koob, 2010) and disinhibiting dopamine neurons (Solinas et al., 2019; Wise and Robble, 2020) in the brain, but chronic use may cause long-term changes in brain regions involved in reward processing (Hyman et al., 2006), leading to escalation of drug intake, elevated drug craving, and uncomfortable tolerance and withdrawal symptoms that can remain a lifelong struggle (Wang, 2019). Beyond the risk of addiction and overdose, parental opioid use could lead to adverse consequences on the offspring’s health. Epidemiological evidence suggests that parental opioid use is associated with lower birth weight (Hunt et al., 2008; Conradt et al., 2019) and adverse outcomes in childhood, such as cognitive deficits (Rubenstein et al., 2019), attention-deficit/hyperactivity disorder (Azuine et al., 2019), susceptibility to the substance of abuse (Abu and Roy, 2021), memory impairment, and difficulties in problem-solving tasks (Sundelin Wahlsten and Sarman, 2013; Konijnenberg and Melinder, 2015). Opioid use and abuse by pregnant women adversely influence intrauterine growth and neurodevelopmental abilities in the offspring (Ross et al., 2015),which is also associated with an increased risk of behavioral disorders later in life (Conradt et al., 2019).

In rodents, a host of studies have demonstrated that maternal exposure to opioids alters anxiety-like behavior (Byrnes et al., 2011), play behavior (Johnson et al., 2011), and rewarding effects (Vassoler et al., 2016) in the offspring. Maternal opioid use is an undeniable concern, yet the impact of opioid use by fathers upon their descendants has not been as widely scrutinized. Recently, preclinical studies have provided evidence that paternal exposure to heroin could result in anxiety-like and aggressive behaviors (Farah Naquiah et al., 2016) in the offspring. Besides, paternal exposure to morphine induces impulsive phenotype (Azadi et al., 2021) in descendants. However, an undeniable concern lies in whether paternal exposure to drug abuse can affect the reward-seeking behavior and response to the substance of abuse in the offspring.

Our previous research provides evidence that paternal cocaine-seeking experience increases vulnerability to developing cocaine addiction-like behavior in their descendants (Le et al., 2017b). Besides, paternal binge-like sucrose consumption causes a decrease in reward-seeking behavior in the offspring (Le et al., 2017a). These findings suggest that paternal reward-seeking experience can alter reward processing in the offspring. However, evidence regarding the effects of paternal opioid exposure on the offspring is still lacking. In the current study, we investigated the effects of paternal chronic heroin self-administration experience on the litter size, body weight, heroin analgesic effect, response to cocaine reinforcement, and other behaviors in the F1 generation.

## Materials and methods

### Animals and housing

All experiments were performed using Sprague Dawley rats (Shanghai Laboratory Animal Center, Chinese Academy of Science) and their progeny. Rats were group-housed at 23°C with a 12 h reverse dark/light cycle (on 19:00 h, off 07:00 h), with access to food and water *ad libitum* before behavioral experiments. Room humidity was controlled at 45%. All the behavioral experiments were carried out using rats aged 8–12 weeks. F0 rats were purchased and habituated to the facility for 1 week before the beginning of any procedure. All animal treatments in this study complied strictly with the National Institutes of Health Guide for the Care and Use of Laboratory Animals. They were also approved by the Animal Care and Use Committee of Shanghai Medical College of Fudan University.

### General procedures of operant conditioning behavior

**Apparatus.** The operant chamber (Med Associates, Inc.) was fitted with a syringe pump and equipped with two cue lights (one blue and another white cue light) above the active and inactive levers and one houselight on the opposite wall, and the rats were housed in sound- and light-proof cubicles. Connected personal computers recorded lever pressing and controlled the presentations of drug injection along with light and tone cues.

**Food training.** To facilitate the acquisition of self-administration behavior, 8-week-old male rats were food restricted and maintained at 85% of their *ad libitum* body weight and were then trained to press the active lever to get food pellets (45 mg, Bio-Serv, Flemington, NJ, United States) in the operant chamber. After successful acquisition of 100 food pellets, the rats were subjected to surgery.

**Surgery.** Under isoflurane anesthesia, the rats were implanted with a chronic indwelling silica jugular catheter (ID = 0.31 mm, OD = 0.64 mm, Dow Corning) into the right jugular vein. The catheter was linked to a printed back-mounted PLA pedestal. After surgery, they were permitted to recover for 7 days, and their catheters were flushed daily with 0.1 mL saline containing heparin (30 IU ml^−1^) and gentamycin (0.5 mg ml^−1^).

**Fixed-ratio reinforcement schedule.** After recovery, the rats were trained to press the lever under a daily 2.5-h FR reinforcement schedule for intravenous drug injection, which consisted of three 40-min drug-available periods separated by two 15-min no-drug periods. During the drug-available periods, when the rats pressed the active lever for a preset number of times, an infusion of drug per injection over 4 s was presented, and a conditioned light–tone cue was accompanied for 20 s. Each infusion was followed by a 20-s time-out period, during which further lever presses were recorded but did not result in any additional injections. However, pressing the inactive lever was recorded but that had no programmed consequences at any time. During the no-drug periods, signaled by the illumination of chamber light and extinction of cue light, pressing the active lever was recorded but resulted in no drug injection or conditioned cue. The maximum number of available drug infusions during the FR reinforcement schedule was 70.

**Progressive-ratio reinforcement schedule.** The rats were tested on a progressive ratio (PR) reinforcement schedule to assess their motivation for drug reinforcement. The 2.5-h PR test was carried out, during which lever press requirements for each successive injection increased by progressive increments. The lever press requirements for each injection (i) follows *i*-th injection = Int (5e^0.25i−5^), and the session stops when the rats take more than 1 h to satisfy the response requirements. Cumulative lever press and breakpoint were recorded during the PR reinforcement schedule. The catheter patency was confirmed after the last PR reinforcement test by using chloral hydrate for anesthesia, and the data of the rats with catheter problems were excluded.

**Intravenous heroin self-administration.** After recovery, the rats were maintained on a 2.5-h daily self-administration training procedure. They were first subjected to self-administration at a fixed ratio of 1 (FR1, one lever press for one injection) for five sessions, then switched to FR5 (five lever presses for one injection) schedule for 25 sessions, and then tested on a progressive ratio (PR) schedule for 1 session. When the rats pressed the active lever during the drug-available periods, an intravenous infusion of heroin at 45 μg kg^-1^ per injection was delivered (50 μL per 4 s).

**Intravenous cocaine self-administration.** After recovery, F1 rats were trained to press the active lever for cocaine under the FR1 reinforcement schedule for three sessions and under the FR5 reinforcement schedule for six sessions. Then, they were switched to the PR reinforcement schedule for one session, as mentioned earlier. When the rats pressed the active lever during the drug-available periods, an intravenous injection of cocaine (Qinghai Pharmaceutical Factory) at 0.75 mg kg^−1^ per injection was delivered (50 μL per 4 s).

### F0 generation and breeding scheme

After 24 hours since the last self-administration session, six rats that self-administered heroin or saline were randomly selected and housed with two naive female rats to generate the F1 generation. In all F1 behavioral tests, 2–3 rats from each litter were randomly selected.

### Body weight measurement

At birth, the litter size and sex ratio of all the litters were. The body weight of the male pups was recorded on postnatal days 4, 6, 10, 17, and 24 during the early juvenile stages. The body weight of 56-day-old F1 generation rats was also recorded before behavioral tests.

### Object-based attention test

The object-based attention test was conducted as described in detail in Alkam et al. (2011) and McCarthy et al. (2018). A two-chambered opaque plexiglass box consisting of an exploring chamber (60 × 40 × 50 cm) and a test chamber (30 × 40 × 50 cm) was used. The rats were acclimated to the environment and experimenters 3 days before the general procedure of the test. Then, on day 4, the rats were placed in the empty exploring chamber for 5 min and then in the test chamber for another 5 min.

The acquisition session began on day 5 by placing each rat in the exploring chamber containing five floor-adhered objects (A, B, C, D, and E), which were made of the same material and were similar in size but of different shapes. Then, each rat was allowed to explore the exploring chamber for 5 min, during which the behavior of the rat was recorded using an overhead camera. Next, the rat was placed in the test chamber containing two objects (A and D) and allowed 5 min for exploring.

On day 6, the rat was first placed in the empty exploring chamber and empty test chamber for 3 min each for habituation. Next, it was returned to the exploring chamber containing the five previously exposed objects for 5 min. After the training session, the rat was moved to the test chamber containing one familiar object, A, and a novel object, F. Object A was placed in a position analogous to its original position in the exploring chamber. The behavior of the rat was recorded using the overhead camera. An investigator blinded to the identity of the rats analyzed the video. A recognition index was calculated using the formula: TN/(TF + TN) × 100, where TF and TN are the time spent during the test session exploring the familiar and novel objects, respectively. The chambers were wiped with 75% alcohol to eliminate any scent clues left by the previous rat.

### Hot plate test

Nociception was assessed using a hot plate test. First, the rats were placed on a thermostable hot plate, with the temperature set to 48°C, 50°C, 52°C, and 54°C incrementally, to assess the basal latency of nocifensive behavior. The latency to lick a hind paw, flick the hind paw, or jump was measured. A 45-s cutoff was established to prevent tissue damage. The rats were removed from the plate immediately upon licking a hind paw or if no response occurred within 45 s. As all rats that were tested at 48°C and 50 °C could stand on the plate for 45 s, and >50% of the rats stayed on the plate for 45 s at 52 °C, the final temperature for heroin-induced analgesic effects was 54 °C. No further increment in temperature was applied to prevent injuries. Then, following a subcutaneous injection of heroin at 1.5 mg kg^−1^, each rat was tested for nocifensive latency at 54 °C on the hot plate at 15 min, 30 min, 60 min, 90 min, and 120 min following the heroin injection. The behavior of the rats was recorded using the overhead camera. An investigator blinded to the identity of the rat analyzed the video recordings to calculate the response latency of each rat. Data from hot plate tests are expressed as percent maximum possible effect (% MPE) = 100% × [(T_test_ − T_baseline_)/(T_cutoff_ − T_baseline_)], where T_test_ is the latency to respond after heroin injection, T_baseline_ is the latency to respond just prior to heroin treatment, and the cutoff is 45 s.

### Statistical analysis

Active lever presses, infusions, inactive lever presses, lever presses during the no-drug period during heroin self-administration, and cocaine self-administration under the FR reinforcement program were analyzed with a mixed linear model with repeated measurements (MMRMs). Cumulative lever presses and % MPE data in the hot plate test were analyzed using the two-way repeated-measures analysis of variance (RM-ANOVA), followed by Bonferroni *post hoc* tests. The breakpoint during the PR reinforcement program was analyzed by the Mann–Whitney rank-sum test. The object-based attention test data were analyzed with Student’s *t*-test. The sample sizes were estimated based on previous experience, and they are similar to those generally employed in the field. *p* < 0.05 was considered statistically significant. Data are presented as mean ± s.e.m.

## Results

### Paternal chronic heroin self-administration experience did not affect body weight of male F1 generation during early juvenile stage or adulthood

**FIGURE 1:**
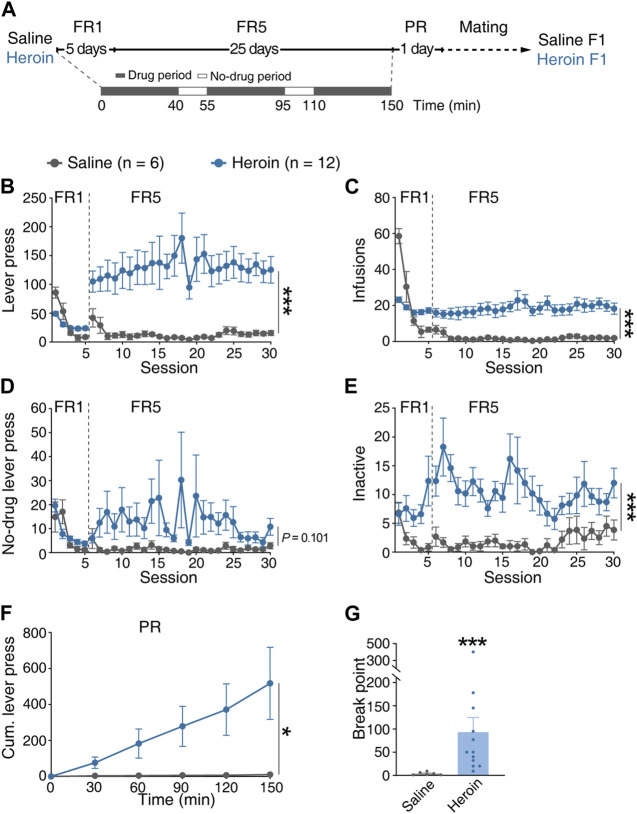
Performance of the F0 generation in heroin self-administration. **(A)** Timeline of the heroin self-administration experiment. The daily 2.5-h FR reinforcement schedule consisted of three 40-min drug-available periods separated by two 15-min no-drug periods. After 31-day heroin or saline self-administration, rats from the heroin or saline groups were mated with naive female rats to generate offsprings. **(B)** Lever press during the FR reinforcement schedule. MMRM, group × FR, χ^2^(1) = 109.64, *p* < 0.001; group, *p* = 0.0703; FR1, *p* = 0.901; FR5, ^***^ *p* < 0.001. **(C)** Infusions during the FR reinforcement schedule. MMRM, group × FR, χ^2^(1) = 120.39, *p* < 0.001; group, *p* = 0.09; FR1, *p* = 0.299; FR5, ^***^ *p* < 0.001. **(D)** Lever press in the no-drug period during the FR reinforcement schedule. MMRM, group × FR, χ^2^(1) = 6.87, *p* = 0.008; group, *p* = 0.386; FR1, *p* = 0.917; FR5, *p* = 0.101. **(E)** Inactive lever press during the FR reinforcement schedule. MMRM, group × FR, χ^2^(1) = 3.39, *p* = 0.065; group, *p* < 0.001; FR1, *p* = 0.012; ^***^FR5, *p* < 0.001. **(F)** Cumulative lever press during the PR reinforcement schedule. Two-way RM-ANOVA, *F*_group×time_(5, 80) = 3.115, *p* = 0.013; group, *p* = 0.104; 120 min, ^*^ *p* = 0.035; 150 min, ^**^ *p* = 0.004. **(G)** Breakpoint during the PR reinforcement schedule. Mann–Whitney rank-sum test, *U* = 0.500, ^***^ *p* < 0.001. Saline, n = 6. Heroin, n = 12. Results are shown as mean ± s.e.m. ^*^ *p* < 0.05, ^**^ *p* < 0.01, ^***^ *p* < 0.001.

To explore the potential effects of paternal chronic heroin self-administration experience on the offsprings, we first randomly assigned a cohort of naive male Sprague Dawley rats to self-administer heroin or saline by pressing the active lever in 2.5-h daily sessions under a fixed-ratio (FR) reinforcement schedule for 30 days and tested on a progressive ratio (PR) reinforcement schedule on day 31 (Figure 1A). We found that under the FR5 reinforcement schedule, the rats from the heroin group (heroin) displayed higher active lever presses (Figure 1B, MMRM, group × FR, χ^2^(1) = 109.64, *p* < 0.001; group, *p* = 0.0703; FR1, *p* = 0.901; FR5, *p* < 0.001) and higher number of infusions (Figure 1C, MMRM, group × FR, χ^2^(1) = 120.39, *p* < 0.001; group, *p* = 0.09; FR1, *p* = 0.299; FR5, *p* < 0.001) than the rats from the saline group (saline). The lever presses of no-drug periods between heroin and saline groups were comparable (Figure 1D, MMRM, group × FR, χ^2^(1) = 6.87, *p* = 0.008; group, *p* = 0.386; FR1, *p* = 0.917; FR5, *p* = 0.101). In addition, the inactive lever presses of the heroin group were significantly higher than those of the saline group (Figure 1E, MMRM, group × FR, χ^2^(1) = 3.39, *p* = 0.065; group, *p* < 0.001; FR1, *p* = 0.012; FR5, *p* < 0.001). Furthermore, the rats from the heroin group showed higher motivation for heroin reward that the rats from the saline group, by displaying higher cumulative lever presses at 120 min and 150 min [Figure 1F, two-way RM-ANOVA, *F*_group×time_(5, 80) = 3.115, *p* = 0.013; group, *p* = 0.104; 120 min, *p* = 0.035; 150 min, *p* = 0.004] and breakpoint (Figure 1G, Mann–Whitney rank-sum test, *U* = 0.500, *p* < 0.001) under the PR reinforcement schedule. After 31-day heroin or saline self-administration, the rats of the heroin or saline group (n = 6 for each group) were mated with naive female rats to generate offsprings.

**FIGURE 2:**
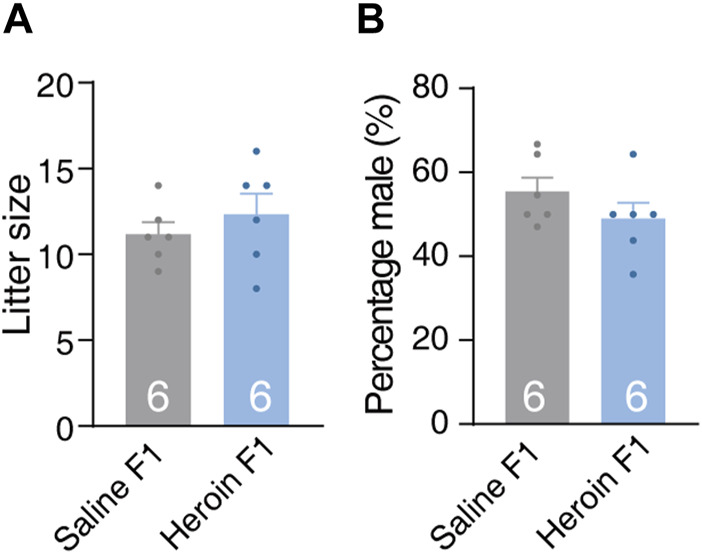
Litter size and sex ratio of F1 offspring. **(A)** Litter size. The number of pups per litter was recorded at birth. Student’s *t*-test, *t*(10) = 0.508, *p* = 0.622. Saline F1, n = 6. Heroin F1, n = 6. **(B)** Sex ratio. The percentage of male pups per litter was recorded at birth. Student’s *t*-test, *t*(10) = 1.272, *p* = 0.232. Saline F1, n = 6. Heroin F1, n = 6. Results are shown as mean ± s.e.m.

We first evaluated the impact of paternal chronic heroin self-administration experience on the litter size, sex ratio, and weight gain trajectory of male offsprings during the early juvenile stage. There was no significant difference in litter size [Figure 2A, Student’s *t*-test, *t* (10) = 0.508, *p* = 0.622] or sex ratio [Figure 2B, Student’s *t*-test, *t* (10) = 1.272, *p* = 0.232] at birth. During the early juvenile stage (postnatal days 4, 6, 10, 17, and 24) and in adulthood (56 days), the body weight of the male F1 offsprings of the heroin group (heroin F1) and male F1 offsprings of the saline group (saline F1) were not different either [Supplementary Figure S1A, two-way RM-ANOVA, *F*_group×day_(5,170) = 0.585, *p* = 0.711]. No difference was observed in female weight gain in adulthood either [Supplementary Figure S1B, Student’s *t*-test, *t* (14) = 0.820, *p* = 0.426]. These data suggest that paternal chronic heroin self-administration experience had no significant effect on weight gain trajectory in the male F1 generation during the early juvenile stage or in adulthood.

### Paternal chronic heroin self-administration experience did not affect cocaine self-administration behavior in F1 generation

**FIGURE 3:**
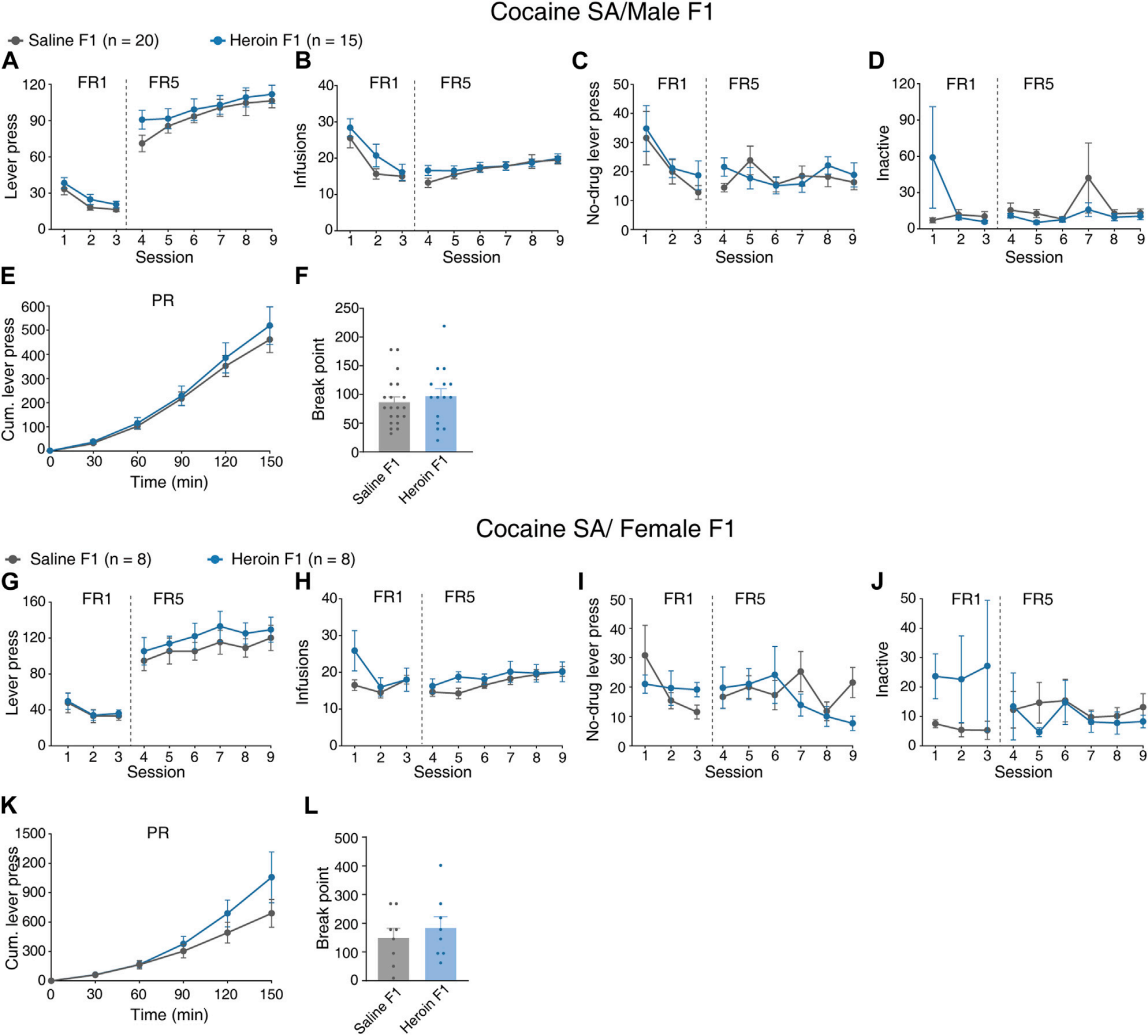
Performance of F1 offspring in cocaine self-administration. **(A–F)** Cocaine self-administration tests in male F1 offspring. **(A)** Lever press during the FR reinforcement schedule. MMRM, group × FR, χ^2^(1) = 0.05, *p* = 0.818; group, *p* = 0.295; FR1, *p* = 0.409; FR5, *p* = 0.265. **(B)** Infusions during the FR reinforcement schedule. MMRM, group × FR, χ^2^(1) = 1.95, *p* = 0.162; group, *p* = 0.207; FR1, *p* = 0.099; FR5, *p* = 0.602. **(C)** Lever press in the no-drug period during the FR reinforcement schedule. MMRM, group × FR, χ^2^(1) = 0.50, *p* = 0.477; group, *p* = 0.577; FR1, *p* = 0.442; FR5, *p* = 0.857. **(D)** Inactive lever press during the FR reinforcement schedule. MMRM, group × FR, χ^2^(1) = 3.54, *p* = 0.059; group, *p* = 0.580; FR1, *p* = 0.147; FR5, *p* = 0.342. **(E)** Cumulative lever press during the PR reinforcement schedule. Two-way RM-ANOVA, *F*_group×time_(5,165) = 0.316, *p* = 0.903; group, *p* = 0.612. **(F)** Breakpoint during the PR reinforcement schedule. Mann–Whitney rank-sum test, *U* = 128.5, *p* = 0.480. Saline F1, n = 20 from six litters. Heroin F1, *n* = 15 from six litters. (**G–L**) Cocaine self-administration tests in female F1 offspring. **(G)** Lever press during the FR reinforcement schedule. MMRM, group × FR, χ^2^(1) = 310.51, *p* = 0.194; group, *p* = 0.518; FR1, *p* = 0.884; FR5, *p* = 0.269. **(H)** Infusions during the FR reinforcement schedule. MMRM, group × FR, χ^2^(1) = 2.30, *p* = 0.513; group, *p* = 0.296; FR1, *p* = 0.185; FR5, *p* = 0.521. **(I)** Lever press in the no-drug period during the FR reinforcement schedule. MMRM, group × FR, χ^2^(1) = 4.68, *p* = 0.197; group, *p* = 0.372; FR1, *p* = 0.101; FR5, *p* = 0.745. **(J)** Inactive lever press during the FR reinforcement schedule. MMRM, group × FR, χ^2^(1) = 12.53, *p* = 0.0058; group, *p* = 0.646; FR1, *p* = 0.010; FR5, *p* = 0.770. **(K)** Cumulative lever press during the PR reinforcement schedule. Two-way RM-ANOVA, *F*_group×time_(5,95) = 1.180, *p* = 0.328, group, *p* = 0.477. (**L**) Breakpoint during the PR reinforcement schedule. Mann–Whitney rank-sum test, *U* = 27.50, *p* = 0.665. Saline F1, *n* = 8 from four litters. Heroin F1, *n* = 8 from four litters. Results are shown as mean ± s.e.m.

We next sought to explore whether paternal chronic heroin self-administration experience affects responses to cocaine reinforcement in the F1 generation. Male heroin F1 rats and saline F1 rats were subjected to food training and surgery and were then trained to self-administer cocaine after recovery. During the 9-day 2.5-h fixed-ratio cocaine self-administration training, the active lever presses of heroin F1 rats were not changed when compared with saline F1 rats [Figure 3A, MMRM, group × FR, χ^2^(1) = 0.05, *p* = 0.818; group, *p* = 0.295; FR1, *p* = 0.409; FR5, *p* = 0.265]. Similar to the active lever presses, the number of infusions of cocaine was comparable between heroin F1 rats and saline F1 rats [Figure 3B, MMRM, group × FR, χ^2^(1) = 1.95, *p* = 0.162; group, *p* = 0.207; FR1, *p* = 0.099; FR5, *p* = 0.602]. Besides, the lever presses of no-drug periods [Figure 3C, MMRM, group × FR, χ^2^(1) = 0.50, *p* = 0.477; group, *p* = 0.577; FR1, *p* = 0.442; FR5, *p* = 0.857] and inactive lever presses [Figure 3D, MMRM, group × FR, χ^2^(1) = 3.54, *p* = 0.059; group, *p* = 0.580; FR1, *p* = 0.147; FR5, *p* = 0.342] were not changed in heroin F1 rats. Under the progressive-ratio paradigm, we found no reliable changes in the cumulative lever presses [Figure 3E, two-way RM-ANOVA, *F*_group×time_(5,165) = 0.316, *p* = 0.903; group, *p* = 0.612] and breakpoint (Figure 3F, Mann–Whitney rank-sum test, *U* = 128.5, *p* = 0.480). Similarly, no significant changes were observed in female offsprings during FR [Figures 3G–J, MMRM, active lever presses, group × FR, χ^2^(1) = 310.51, *p* = 0.194, group, *p* = 0.518; infusions, group × FR, χ^2^(1) = 2.30, *p* = 0.513, group, *p* = 0.296; no-drug lever pressed, group × FR, χ^2^(1) = 4.68, *p* = 0.197, group, *p* = 0.372; inactive lever press, group × FR, χ^2^(1) = 12.53, *p* = 0.0058, group, *p* = 0.646] or PR sessions [Figure 3K, L, cumulative lever press during PR, two-way RM-ANOVA, *F*_group×time_(5,95) = 1.180, *p* = 0.328, group, *p* = 0.477; breakpoint, Mann–Whitney rank-sum test, *U* = 27.50, *p* = 0.665]. These results suggest that paternal chronic heroin self-administration experience may not affect the responses to cocaine reinforcement in F1 offsprings.

### Paternal chronic heroin self-administration experience had no significant impact on object-based attention behavior in F1 generation

**FIGURE 4:**
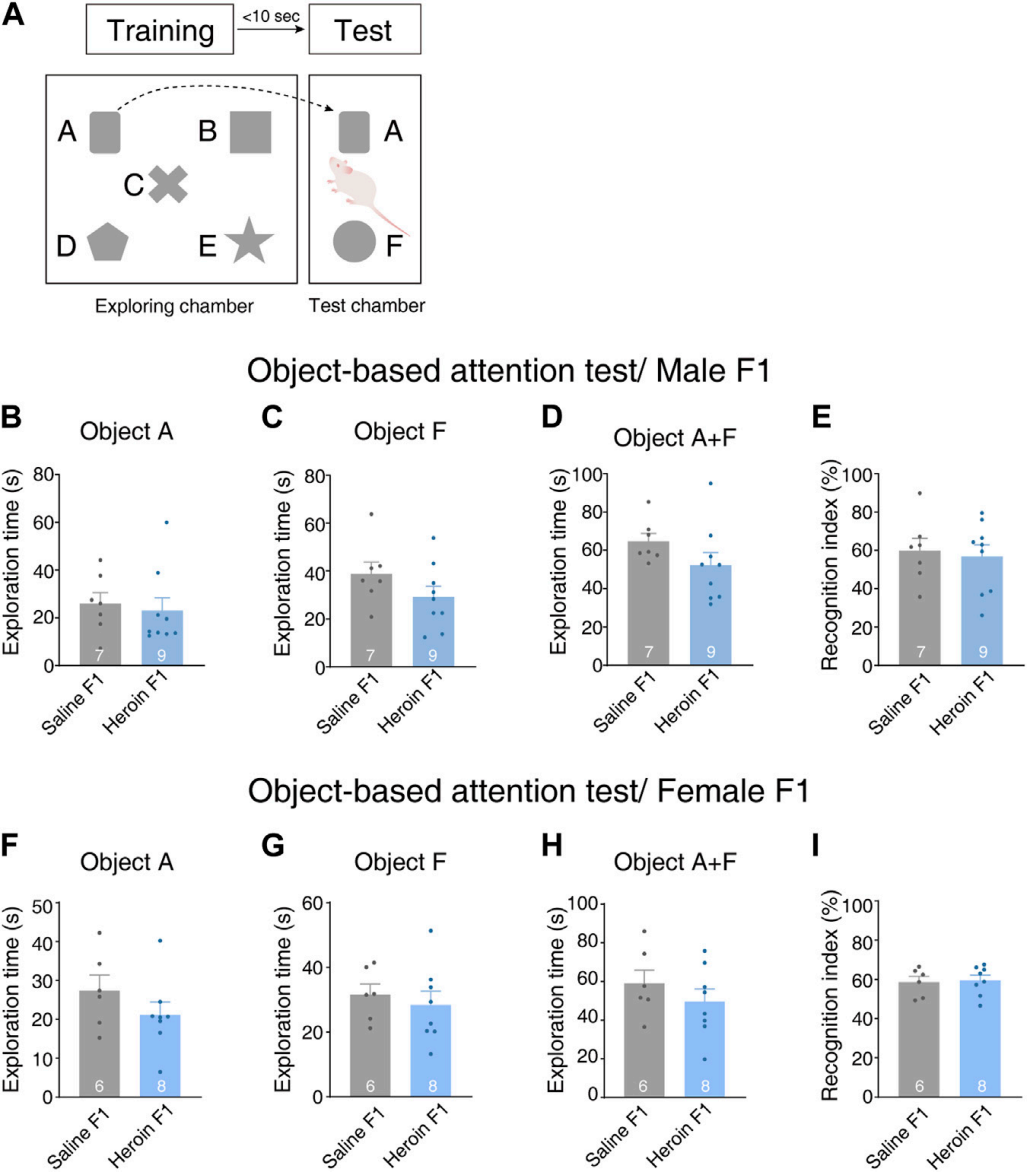
Performance of F1 offspring in the object-based attention test. **(A)** Experimental design. Five objects **(A–E)** were floor fixed and placed separately in the center arena of the exploring chamber. Rats were first allowed to explore the objects in the exploring chamber. Then, one object used in the training session was placed into the test chamber in parallel with its original position in the exploring chamber. A novel object F in a different shape but of similar color and size was placed in the test chamber. Then, the rats were allowed to enter the test chamber and explore two objects: the familiar object A and the novel object F. **(B–E)** Object-based attention test in male F1 offspring. **(B)** Object A exploration time. Student’s *t*-test, *t*(14) = 0.393, *p* = 0.699. **(C)** Object F exploration time. Student’s *t*-test, *t*(14) = 1.431, *p* = 0.174. **(D)** Total exploration time. Student’s *t*-test, *t*(14) = 1.489, *p* = 0.158. **(E)** Recognition index. The recognition index, calculated for each rat, was expressed as the ratio (TF × 100)/(TA + TF), where TA and TF are the time spent on object A and object F, respectively. Student’s *t*-test, *t*(14) = 0.350, *p* = 0.731. Saline F1, n = 7 from five litters. Heroin F1, n = 9 from five litters. **(F–I)** Object-based attention test in female F1 offspring. **(B)** Object A exploration time. Student’s *t*-test, *t*(12) = 1.193, *p* = 0.256. **(C)** Object F exploration time. Student’s *t*-test, *t*(12) = 0.555, *p* = 0.589. **(D)** Total exploration time. Student’s *t*-test, *t*(12) = 0.970, *p* = 0.351. **(E)** Recognition index. Student’s *t*-test, *t*(12) = 0.225, *p* = 0.826. Saline F1, n = 6 from four litters. Heroin F1, n = 8 from four litters. Results are shown as mean ± s.e.m.

To examine the effect of paternal chronic heroin self-administration experience on cognitive function in the F1 generation, we conducted an object-based attention experiment. On the test day, the rats were first allowed to freely explore five objects that were different in shape. After the exploration session, they were moved to the test chamber to explore one of the familiar objects, object A, placed in a position analogous to its original position in the exploring chamber, and a novel object, object F, to which the rat had never been exposed (Figure 4A). We recorded the exploration time of the familiar object A and novel object F. In male offsprings, there was no difference in time spent exploring object A [Figure 4B, Student’s *t*-test, *t*(14) = 0.393, *p* = 0.699] or F [Figure 4C, Student’s *t*-test, *t*(14) = 1.431, *p* = 0.174] between heroin F1 rats and saline F1 rats. Furthermore, we did not observe any change in total exploration time either [Figure 4D, Student’s *t*-test, *t*(14) = 1.489, *p* = 0.158]. The recognition index of heroin F1 rats was comparable to that of saline F1 rats [Figure 4E, Student’s *t*-test, *t*(14) = 0.350, *p* = 0.731]. No significant difference was observed in female F1 rats either [Figures 4F–I, Student’s *t*-test, object A exploration time, *t*(12) = 1.193, *p* = 0.256; object F exploration time, *t*(12) = 0.555, *p* = 0.589; total exploration time, *t*(12) = 0.970, *p* = 0.351; recognition index, *t*(12) = 0.225, *p* = 0.826]. These results suggest that paternal chronic heroin self-administration experience has no significant impact on object-based attention behavior in the F1 generation.

### Paternal chronic heroin self-administration experience increased analgesic effect of heroin in male F1 generation

**FIGURE 5:**
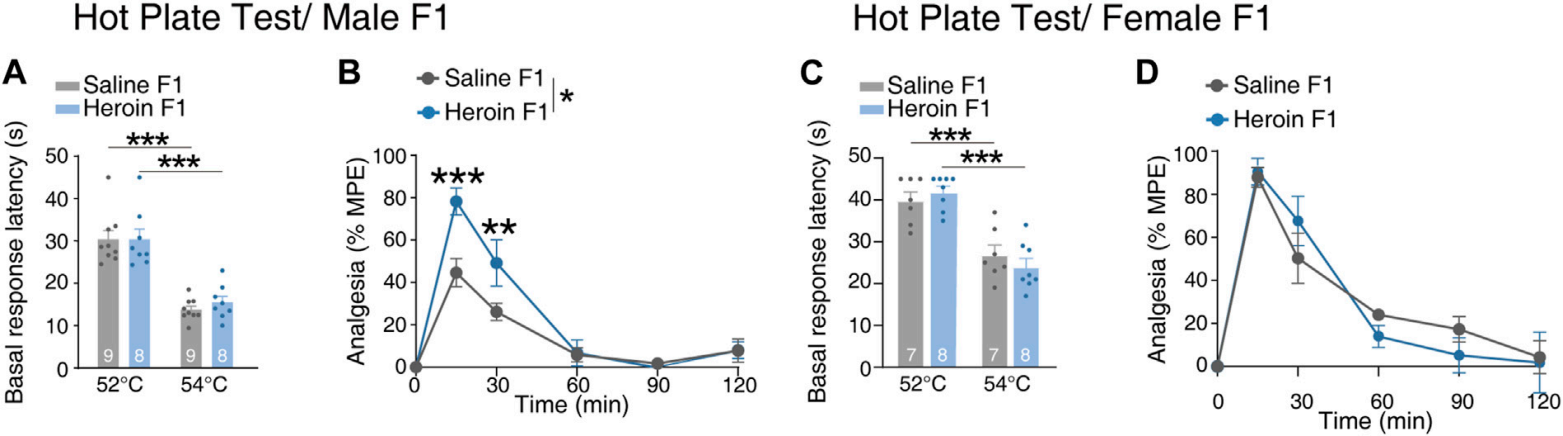
Performance of F1 offspring in the heroin-induced anti-nociception test. **(A, B)** The hot plate test in male F1 offspring. **(A)** Basal response latency. Two-way RM-ANOVA, *F*_group×temperature_(1,15) = 0.219, *p* = 0.647; temperature, *p* < 0.001; group, *p* = 0.618; 52°C, *p* = 0.996; 54°C, *p* = 0.496. **(B)** Heroin analgesia. Two-way RM-ANOVA, *F*_group×time_(5,75) = 4.60, *p* = 0.001; group, ^*^ *p* = 0.049; 15 min, ^***^ *p* < 0.001; 30 min, ^**^ *p* = 0.004. Saline F1, n = 9 from six litters. Heroin F1, *n* = 8 from six litters. **(C, D)** The hot plate test in female F1 offspring. **(C)** Basal response latency. Two-way RM-ANOVA, *F*_group×temperature_(1,13) = 2.294, *p* = 0.154; temperature, *p* < 0.001; group, *p* = 0.856; 52°C, *p* = 0.949; 54°C, *p* = 0.627. **(D)** Heroin analgesia. Two-way RM-ANOVA, *F*_group×time_(5,60) = 0.754, *p* = 0.587; group, *p* = 0.742. Saline F1, n = 6 from four litters. Heroin F1, n = 6 from four litters. Results are shown as mean ± s.e.m. ^*^ *p* < 0.05, ^**^ *p* < 0.01, ^***^ *p* < 0.001.

To investigate if paternal chronic heroin self-administration experience could affect the response to heroin in the F1 generation, we conducted a hot plate experiment. First, to evaluate the pain perception to the thermal stimulus of the male F1 generation, we recorded the basal response latency when the rats were placed on a 52°C or 54°C plate. The basal response latency of rats on the 54°C plate was significantly lower than that for rats on the 52°C plate, but there was no difference in the basal response latency between heroin F1 rats and saline F1 rats when they were placed on 52°C or 54°C plates [Figure 5A, two-way RM-ANOVA, *F*_group×temperature_(1,15) = 0.219, *p* = 0.647; temperature, *p* < 0.001; group, *p* = 0.618; 52°C, *p* = 0.996; 54°C, *p* = 0.496]. Thus, as latencies measured at 54°C was within a reasonable range (10–30 s). We chose to use the 54°C plate to assess the analgesic effect of heroin in the male F1 generation. We found that the maximum possible effect after heroin injection of heroin F1 rats was significantly higher than that of saline F1 rats, especially at 15 min and 30 min [Figure 5B, two-way RM-ANOVA, *F*_group×time_(5,75) = 4.60, *p* = 0.001; group, *p* = 0.049; 15 min, *p* < 0.001; 30 min, *p* = 0.004]. On the contrary, no significant difference was observed in female F1 rats, either under basal response latency tests [Figure 5C, two-way RM-ANOVA, *F*_group×temperature_(1,13) = 2.294, *p* = 0.154; temperature, *p* < 0.001; group, *p* = 0.856] or in heroin-induced analgesia [Figure 5D, two-way RM-ANOVA, *F*_group×time_(5,60) = 0.754, *p* = 0.587; group, *p* = 0.742]. These results suggest that paternal chronic heroin self-administration experience significantly increased the analgesic effect of heroin specifically in male offsprings but did not alter the pain perception to thermal stimulus in either male or female offsprings.

## Discussion

In the current study, we assessed the impact of paternal chronic heroin self-administration experience on reward and cognitive behaviors in offsprings. Our results have suggested that paternal chronic heroin self-administration experience had no significant impact on weight gain trajectory during the early juvenile stage or in adulthood in male offsprings. In addition, our results have indicated that paternal chronic heroin self-administration experience did not exert observable influence on cocaine self-administration or object-based attention behaviors in the F1 offspring. Furthermore, we found that paternal chronic heroin self-administration experience did not alter the pain perception to thermal stimulus in the F1 generation but significantly increased the analgesic effect of heroin in male offsprings.

It is an interesting and frequently observed phenomenon that most of the influences of ancestral exposure to drugs of abuse are sex specific (Pierce et al., 2018). Recent evidence has reported a dichotomy in the phenotypic changes resulting from parental drug exposures dependent on the sex of offsprings. For example, paternal morphine self-administration selectively disrupted novel object recognition in the female but not male progeny (Ellis et al., 2020). Decreased cocaine self-administration behavior was observed in the male but not female offspring of sires exposed to cocaine (Vassoler et al., 2013). Morphine exposure prior to conception exerted sex-specific alterations in endogenous opioids and hypothalamic physiology (Vassoler et al., 2019). To date, the study of transgenerational effects of addictive drugs is still in its early stages, and several possible explanations have been raised. First, regarding the mechanism of transgenerational inheritance, there is evidence that drug abuse or the drug itself can modify epigenetic marks in the germline, affecting mitochondrial genes or genes found on the Y-chromosome, and genomic imprinting may also play a role. Moreover, there is a natural sex disparity in the prevalence of drug addiction. Biological sex is known to dictate drug-related behavioral and neurobiological outcomes (Becker et al., 2012). Women are shown to exhibit more rapid escalation from causal drug taking to addiction than men (Bobzean et al., 2014), and previous rodent studies (Cicero et al., 2003) and our data (Figure 3) both suggest females showing more rapid acquisition of heroin-taking behavior and greater overall intake. Sex differences, both at molecular and circuit levels, were observed in key nuclei involved in addiction, like the bed nucleus of the stria terminalis (Chung et al., 2000) and the ventral tegmental area (McArthur et al., 2007). In this study, we observed male-specific increase in the analgesic effect of heroin caused by paternal chronic heroin self-administration experience. Novel methods to accurately trace the influence of germline modifications through embryogenesis and development will be crucial for advancing the field and developing effective interventions to mitigate the negative effects of drug exposure on future generations.

Epidemiological evidence suggests that parental opioid exposure is associated with lower birth weight and adverse developmental outcomes in offsprings (Hunt et al., 2008). Preclinical research found that maternal exposure to opioids from conception through the pup weaning process results in reduced body weight in the offsprings (Minakova et al., 2021). By contrast, other research has indicated that maternal exposure to oxycodone did not affect the birth weight or body weight on postnatal day 8, day 21, and day 30 (Minakova et al., 2022). However, few studies have investigated the effect of paternal chronic exposure to opioids on the weight gain trajectory during the early juvenile stage and in adulthood in offsprings. In this study, we recorded the body weight of the male F1 generation and did not find any difference in body weight between male heroin F1 rats and saline F1 rats on postnatal days 4, 6, 10, 17, or 24. Besides, the body weight of 56-day-old heroin F1 rats was comparable to that of saline F1 rats. These results suggest that paternal chronic heroin self-administration experience may not influence the weight gain trajectory during the early juvenile stage or in adulthood in offsprings.

Less is known regarding how exposure to one drug in the F0 generation may impact response to a different drug of abuse in the F1 generation. Previous studies have found that parental exposure to THC decreases motivation to self-administer heroin in the male F1 generation (Hempel et al., 2020). Maternal exposure to morphine increased motivation to self-administer cocaine in male and female progeny (Vassoler et al., 2019). However, in another work, paternal cocaine exposure had no effect on nicotine self-administration in the F1 generation (Wimmer et al., 2019). In our study, no differences were observed during the cocaine acquisition phase (FR1 and FR5 reinforcement schedule). Besides, there was no difference in breakpoint in the PR reinforcement schedule between the two groups, suggesting the motivated response for cocaine reinforcement was not changed in heroin F1 rats. There are overlapping and discrete mechanisms underlying the neuronal adaptions in response to different drugs of abuse, both in drug-exposed individuals and their offsprings. An increase in dopamine levels in the mesolimbic system is hypothesized as the initial pharmacological effect common to all addictive drugs (Luscher and Ungless, 2006; Luscher and Janak, 2021), but this effect is mediated by divergent mechanisms of action distributed across different brain regions and cell types (Badiani et al., 2011; Browne et al., 2020). A recent study has provided evidence that paternal exposure to morphine increases the expression of κ-opioid receptor transcript in the prefrontal cortex in the offspring, reducing nociception in male offsprings (Ashabi et al., 2018). In addition, parental THC exposure induced molecular and synaptic changes in dopaminergic neurons and elicited behavioral susceptibility to THC in male offsprings (Frau et al., 2019). To better interpret the drug-specific or cross-sensitization effects on offsprings, it appears that comprehensive neurocircuitry studies, possibly with a focus on the midbrain mesolimbic region, are required. Besides, it is worth pointing out that the sex of the parent, the time between drug exposure and mating, i.e., the period of abstinence, and the mode of drug delivery in the sires (contingent vs. non-contingent) seem to play important roles in influencing the behavior and biology in the next generation.

The current, yet limited, clinical literature evaluating the effects of parental exposure to opioids on children suggests an increased incidence of attention-deficit hyperactivity disorder (Ornoy et al., 2001; Schwartz et al., 2021). A previous study also demonstrated that paternal exposure to nicotine produces significant deficits in attention and reversal learning of the male F1 generation (McCarthy et al., 2018). However, few studies have investigated the effect of parental chronic exposure to opioids on attention behavior in offsprings. We used the object-based attention test for the analysis of attention in the F1 generation. In our study, the total exploration time was similar, and both saline F1 and heroin F1 rats spent more time exploring the novel object, as revealed by the recognition index, suggesting that paternal chronic heroin self-administration experience had no significant impact on object-based attention behavior in the offspring. However, more rigid assays are required to completely exclude the potential effects of paternal chronic heroin-seeking experience on the attention behavior in offsprings.

Opioids are known to be potent analgesic drugs. In the current study, we found that the basal thermal pain threshold was comparable between heroin F1 and saline F1 generations when the rats were placed on a 52°C or 54°C plate, suggesting that the pain perception to the thermal stimulus was not changed in heroin F1 rats. However, an acute injection of heroin (1.5 mg kg^−1^) significantly increased the maximum possible effect of heroin F1 rats when compared with saline F1 rats. A previous study also demonstrated that maternal exposure to opioids increased sensitivity to the analgesic effects of acute morphine in male offsprings (Byrnes et al., 2011). Furthermore, there is a strong association between analgesic effect and abuse liability (Franklin, 1989; Franklin, 1998). These data suggest that paternal chronic heroin self-administration experience may increase the risk of drug abuse vulnerability in male offsprings. However, the correlation between paternal chronic heroin self-administration experience and substance abuse liability in offsprings warrants future study.

## Data Availability

The raw data supporting the conclusion of this article will be made available by the authors, without undue reservation.
