# Supplementary figures and images for: Enhanced heroin analgesic effect in male offspring of sires who self-administered heroin
Source: Front Pharmacol. 2023 Jun 14;14:1211897. doi: 10.3389/fphar.2023.1211897 (PMC10303812; doi:10.3389/fphar.2023.1211897)

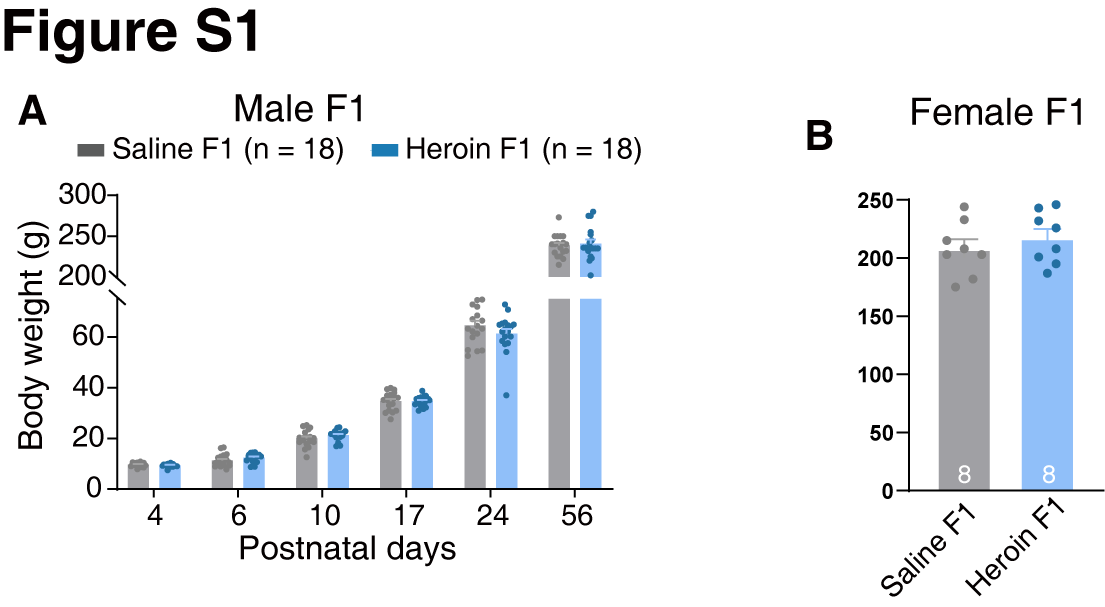

Supplement: Supplementary file 1 [file Image1.TIF]
